# Insights from BRICS-T economies on the impact of human capital and renewable electricity consumption on environmental quality

**DOI:** 10.1038/s41598-023-32134-1

**Published:** 2023-03-31

**Authors:** Ahmed Samour, Tomiwa Sunday Adebayo, Ephraim Bonah Agyekum, Baseem Khan, Salah Kamel

**Affiliations:** 1 Accounting Department, Dhofar University, Salalah, Sultanate of Oman; 2 Department of Economics, Faculty of Economics and Administrative Sciences, Cyprus International University, Mersin 10, 99040 Haspolat, Turkey; 3 Department of Nuclear and Renewable Energy, Ural Federal University Named After the First President of Russia Boris Yeltsin, 19 Mira Street, Ekaterinburg, Russia 620002; 4 Department of Electrical and Computer Engineering, Hawassa University, Hawassa, Ethiopia; 5 Electrical Engineering Department, Faculty of Engineering, Aswan University, Aswan, 81542 Egypt

**Keywords:** Electrical and electronic engineering, Energy infrastructure

## Abstract

This paper evaluates the impact of electricity consumption from renewable and nonrenewable sources on the load capacity factor for BRICS-T nations using data from 1990 to 2018. The paper used linear and nonlinear autoregressive distributed lag (ARDL) approaches to explore these associations. The results of the Westerlund co-integration show long-run co-integration between load capacity factor and the independent variables. The results show that renewable electricity energy and human capital contribute to the sustainability of the environment, while electricity consumption, economic growth, and industrialization impede environmental sustainability. Similarly, the nonlinear effect of renewable electricity energy on LCF shows interesting findings. The positive (negative) shift in renewable electricity energy increases ecological sustainability in the BRICS-T nations. Furthermore, the Dumitrescu Hurlin panel causality gives credence to both linear and nonlinear ARDL results. The study suggests policy recommendations based on these results.

## Introduction

In the last decades, environmental quality issue has become the main goal for most developing and developed nations^1,2^. Environmental sustainability includes all the practices and activities that reinforce long-term economic development without adversely impacting society^3,4^. Environmental sustainability can only be achieved by exploring the linkage between growth and environmental pollution through industrialization. This linkage demonstrates that industrialisation is influential in accelerating economic development activities, which eventually negatively affect environmental sustainability by increasing energy demand and consumption. Various empirical studies support a positive linkage between industrialisation and carbon emissions^5^^,^^6^^,^^7^.

However, global warming is significantly attributed to economic growth, where economic expansion is prioritized at the cost of ecological sustainability. Over the years, emerging economies such as BRICS-T nations have followed the pro-growth agenda with little attention to environmental sustainability. The economic progress in BRICS-T is highly dependent on energy use as it significantly influences economic growth^10^. The energy demand in the BRICS-T nations has increased considerably over the last decades, which several reasons, such as the level of population, urbanization, and industrialization can explain. However, many policymakers and environmental scholars have highlighted this economic progress's adverse effect on these nations' environmental quality^8,9^. In this regard, the BRICS-T states are ranked among high-polluting economies, particularly China, India, Turkey, and Russia, among the top ten polluting economies in the world. However, the rapid financial and economic development of the BRICS-T nations has several socioeconomic advantages, such as industrialization and infrastructure development, reeducation, reduction in poverty, and increased employment rates in these nations. However, the impact of economic progress and industrialization on ecological sustainability still needs to be investigated^10^.

Achieving ecological sustainability will not be easy if human capital is not considered. Human capital is the combination of distinct parts of competencies, abilities, skills, and knowledge obtained by persons during their daily lifetime, created by communicating in different types of training and education. Therefore, this type of capital aims to enhance employees' knowledge, skills, communications and social values, which will positively affect the society^11,12^. Human capital may affect ecological quality in three ways. First, human capital has a powerful effect on economic and financial expansion; and as a result, may affect energy utilization and the environment. Second, human capital may affect the environment through green technology channels. This way, improving human capital will enhance research, development, and green technologies. Thirdly, human capital may affect the environmental quality by motivating the people and market to preserve the natural environment and promote green consumption and production, leading to a sustainable environment.

In the framework of the BRICS-T, studies employing load capacity factor (LCF) as an ecological sustainability proxy have yet to be adequately employed by the available panel studies. As a result, to fill up this crucial research gap, the paper used the nonlinear ARDL approach to examine the interconnection among the chosen variables. Thus, the present analysis explores the effect of electricity consumption from renewable and non-renewable, human capital, industrialisation and economic growth on load capacity factor. The current investigation is particularly unique in that it provides policy insights for the BRICS-T nations. At the same time, previous studies used CO_2_ and ecological footprint as proxies of ecological degradation, which is not a comprehensive measure of environmental quality/degradation. As a result, we used the LCF, which considers the supply and demand sides of the ecosystem, to explore the effect of electricity consumption from renewable and non-renewable energy, human capital, and industrialisation on LCF in BRICS-T economies from 1990 to 2018. The primary research questions of the present empirical investigation are mentioned below:

- Does a positive (negative) shift in electricity consumption from renewable energy promote ecological sustainability?
- Do industrialisation and economic growth contribute to ecological degradation in BRICS-T nations?
- Does human capital contribute to BRICS-T nations ecological sustainability
- Does electricity consumption from non-renewable energy promote ecological sustainability?

The current investigation contributes to ongoing literature in two ways: I) To the researcher's knowledge, no research has assessed the impact of industrialisation and human capital on ecological sustainability using LCF as a proxy of environmental quality. Thus, we fill the gap in the literature. II) The current analysis explores the asymmetric linkage between electricity consumption from renewable energy and LCF using the nonlinear ARDL approach by^13^. The benefit of this technique is that it captures both (positive/negative) shift effects of the independent variable on the dependent variable.

The subsequent structure of the current investigation is as follows: the second part portrays the literature review followed by the tested method framework. The fourth section describes empirical outcomes and discussion, while the last section shows the conclusion of the current work.

## Literature review

The main aim of policymakers, governments and scholars is to investigate the drivers of environmental quality. Scholars have highlighted several determinants of environmental quality using different countries/nations, technique(s), and timeframe. Nonetheless, varied results have surfaced based on the reasons mentioned earlier. Economic progress is one of the main drivers of ecological quality. Earlier studies such as^14–19^^20^^,^^21^ on the association between economic expansion and environmental deterioration reported that an upsurge in GDP causes deterioration in the ecosystem. A similar report is documented by the study of^22^ on the connection between real growth and ecological quality. The research report uncovered that the pro-growth policies of BRICS trigger the deterioration of the ecosystem. Based on the above knowledge, the following hypothesis is crafted.

*H1*: Economic growth does not improve environmental sustainability.

Regarding the nexus between energy (renewable (REC) and non-renewable (NREC)) and environmental quality, extensive studies have been documented. The study of^23^ in China on the role of energy towards ecological neutrality using data from 1990Q1 and 2018Q4 reported that both fossil fuel and renewable energy contribute to decreased ecological integrity. Contrarily, the paper of^24^ in Thailand on the role of energies from 1980 to 2018 reported that NREC escalates CO_2_ while REC mitigates the emission of CO_2_. Likewise,^25^ inspected the energies-emissions association in the USA using the ARDL. The results gathered from their analysis reported that environmental sustainability is achieved via using REC while NREC lessens the EQ. Recently, in their papers on the energies-environment connection, for the case of OECD and BRICS economies^26^ and^27^ reported that an increase in REC mitigates environmental pollution. Based on the above knowledge, the following hypothesis is crafted.

*H2*: Renewable electricity consumption does not improve environmental sustainability.

*H3*: Non-renewable electricity consumption does not improve environmental sustainability.

Human capital is tied to education, enhanced awareness, knowledge access, and other variables that assist in lowering CO_2_ emissions through implementing appropriate environmental protection policies. Thus, while analysing CO_2_ emissions, human capital is a key explanatory factor. The^28^ documented that an upsurge in human capital triggers ecological integrity. The study of^29^ scrutinized Pakistan's human capital-emissions link between 1980 and 2014. Results from this study disclosed that an intensification of human capital causes ecological sustainability in Pakistan. Based on the above knowledge, the following hypothesis is crafted.

*H4*: Human Capital does not improve environmental sustainability.

Studies on the nexus between industrialisation and ecological deterioration have recently received considerable attention. For illustration,^30^ inspected the nexus between industrialisation and the environment in Sub-Saharan African countries between 1996 and 2019. The study result shows that an upsurge in industrialisation causes CO_2_ emissions intensification. Using N-11 countries, reference^31^ study on the emissions-industrialization using panel ARDL documented positive emissions-industrialization. Contrarily, the work of^32^ in Pakistan using data from 1990 to 2018 reported a negative and insignificant nexus between CO_2_ and industrialisation using the ARDL. Similarly, the study of^33^ in China revealed that an upsurge in industrialisation causes deterioration in the ecosystem. Based on the above knowledge, the following hypothesis is crafted.

*H5*: Industrialisation does not improve environmental sustainability.

Considering the empirical studies mentioned above, it is clear that the influence of electricity consumption from renewable and non-renewable on LCF has yet to be investigated. Thus, the current work fills the gap in environmental and energy literature. The existing investigation is unique in providing policy insights for the BRICS-T nations. At the same time, prevised studies employed CO_2_ and ecological footprint as proxies of ecological degradation/quality, which is not a comprehensive measure of environmental assessment. As a result, we used the LCF to study the effect of electricity consumption from renewable and non-renewable energy, human capital, and industrialization on LCF in BRICS-T states.

## Data and methods

### Data

The study evaluates the effect of ELE and RECE on ecological neutrality in BRICS-T states. The study used other environmental quality drivers such as economic expansion, industrialisation and human capital. The research period encompasses the period between 1990 and 2018. The dependent variable is LCF, estimated as a division of biocapacity and ecological footprint (Global hectares per/capita). The regressors are electricity consumption (ELE) from non-renewable, electricity consumption (RECE) from renewable, which is calculated as GWh, and economic growth (EG), which is captured as GDP Per/capita constant USD-2010, industrialisation (IND) which is measured in constant 2010-US$, and human capital (HC) which is based on schooling years and returns to education. Furthermore, EG and IND are collected from the WB database, ELE and RECE are sourced from the database of BP, LCF is gathered from the database of the global footprint website, and HC is obtained from PENN World Database. İn line with the study of^22^, the study economic model is proposed by incorporating electricity consumption from renewable and human capital as follows:

1 $${LF}_{t}={\mu }_{0}+{\mu }_{1}{EG}_{it}+{\mu }_{2}{ELE}_{it}+{\mu }_{3}{RECE}_{it}+{\mu }_{4}{IND}_{t}+ {\mu }_{4}{HC}_{it}+{z}_{it}$$

where; EG, RECE, ELE, HC, IND, and LCF represent economic growth, electricity consumption from renewables, and non-renewable, human capital, industrialisation and LCF.

### Methods

To inspect the long- and short-term effects of ELE, RECE, IND, and HC on ecological quality in BRICS-T nations, the research utilised panel nonlinear ARDL (PNARDL). Moreover,^35^ proposed the Panel ARDL testing paradigm, which enables the discovery of long and short-term associations. This technique estimates the tested model with I(1) or I(0) series, or both I(1) and I(0). On the flip side, this strategy offers effective and reliable results since it avoids the problems brought on by endogeneity. The model can also be used with small data sample. The ARDL testing technique (p, q) is outlined as follows in line with the panel ARDL model proposed by^34^.

2 $${\mathrm{y}}_{\mathrm{it}}= {\upbeta }_{0} +\mathrm{ \vartheta i}\sum_{\mathrm{j}=1}^{\mathrm{p}}{\mathrm{Y}}_{\mathrm{it}-\mathrm{j}}+\mathrm{ \varnothing i}\sum_{\mathrm{j}=1}^{\mathrm{q}}{\Delta \mathrm{X}1}_{\mathrm{it}-\mathrm{j}}+\mathrm{\varnothing i}\sum_{\mathrm{j}=1}^{\mathrm{q}}\Delta {\mathrm{L}2}_{\mathrm{it}-\mathrm{j}}+\mathrm{\varnothing i}\sum_{\mathrm{j}=1}^{\mathrm{q}}\Delta {\mathrm{M}3}_{\mathrm{it}-\mathrm{j}}+{\uplambda 1\mathrm{ Y}}_{\mathrm{it}-1}+{\uplambda 2\mathrm{X}}_{\mathrm{it}-1}+\uplambda {3\mathrm{L}}_{\mathrm{it}-1}+\uplambda {4\mathrm{ M}}_{\mathrm{it}-1}+\uplambda {5\mathrm{N}}_{\mathrm{it}-1}+\mathrm{ \varphi }{\mathrm{ECT}}_{\mathrm{t}-1}+{\varepsilon }_{it}$$

Where; the endogenous variable is depicted by ${\mathrm{y}}_{\mathrm{it}}$ and the regressors are denoted by $\mathrm{X},\mathrm{L},\mathrm{M},\mathrm{N}$. Moreover, the difference operator is denoted by ∆, error term is illustrated by ε it, and ECT shows error correction term. The long and short-run coefficients are illustrated by Λ1, λ2, λ3, λ4, λ5 and $\mathrm{\varnothing }1,\varnothing 2,\varnothing 3,\mathrm{\varnothing }4,\mathrm{\varnothing }5$, respectively. In addition, optimal lag length is denoted by p, q. The linear panel ARDL is depicted as follows;

3 $${\mathrm{LCF}}_{\mathrm{it}}= {\upbeta }_{0} +\mathrm{ \vartheta }1\sum_{\mathrm{j}=1}^{\mathrm{p}}{\mathrm{LCF}}_{\mathrm{it}-\mathrm{j}}+\mathrm{\varnothing }2\sum_{\mathrm{j}=0}^{\mathrm{q}}{\Delta \mathrm{GDP}}_{\mathrm{it}-\mathrm{j}}+\varnothing 3\sum_{\mathrm{j}=0}^{\mathrm{q}}\Delta {\mathrm{RECE}}_{\mathrm{it}-\mathrm{j}}+\mathrm{\varnothing }4\sum_{\mathrm{j}=0}^{\mathrm{q}}\Delta {\mathrm{ELE}}_{\mathrm{it}-\mathrm{j}}+\mathrm{\varnothing }5\sum_{\mathrm{j}=0}^{\mathrm{q}}{\mathrm{IND}}_{\mathrm{it}-\mathrm{j}}+\mathrm{\varnothing }6\sum_{\mathrm{j}=0}^{\mathrm{q}}{\mathrm{HC}}_{\mathrm{it}-\mathrm{j}}+\uplambda {1\mathrm{ LCF}}_{\mathrm{it}-1}+{\uplambda 2\mathrm{GDP}}_{\mathrm{it}-1}+\uplambda {3\mathrm{RECE}}_{\mathrm{it}-1}+\uplambda {4\mathrm{ELE}}_{\mathrm{it}-1}+\uplambda {5\mathrm{IND}}_{\mathrm{it}-1}+\uplambda {6\mathrm{IND}}_{\mathrm{it}-1}+\mathrm{\varphi }{\mathrm{ECT}}_{\mathrm{it}-1}+ {\varepsilon }_{it}$$

where: the endogenous variable is denoted by ${LCF}_{it}$, the regressors are depicted by $GDP,RECE,ELE, IND,HC$, ∆ and *ε* _it_ representing difference operator and error term, respectively. The long-run coefficients are illustrated by Λ1, λ2, λ3, λ4, λ5 and λ6. In addition, the short-run coefficients are depicted by $\mathrm{\varnothing }1,\varnothing 2,\mathrm{\varnothing }3,\mathrm{\varnothing }4,\varnothing 5\mathrm{ and},\varnothing 6$ while the *optimal* of lag length is shown by (*p, q).*

The modified NARDL testing strategy recommended by^13^ was used in the current work. The primary benefit of this method is that it evaluates the asymmetric impact of the regressor on the endogenous variable. Regressors are divided into positive and negative alterations by the NARDL. To capture the asymmetric estimates, the primary variable, which is electricity consumption from renewable energy, is divided into two parameters, i.e., electricity consumption from renewable energy in two parts. They are the positive and negative shifts in electricity consumption from renewable energy, which is shown as follows in Eq. (4):

4 $${\mathrm{LCF}}_{\mathrm{it}}= {\upbeta }_{0} +\mathrm{ \vartheta }1\sum_{\mathrm{j}=1}^{\mathrm{p}}{\mathrm{LCF}}_{\mathrm{it}-\mathrm{j}}+\varnothing 2\sum_{\mathrm{j}=0}^{\mathrm{q}}{\Delta \mathrm{GDP}}_{\mathrm{it}-\mathrm{j}}+\varnothing 3\sum_{\mathrm{j}=0}^{\mathrm{q}}{\Delta \mathrm{RECE}}_{it-j}^{+}+\varnothing 4\sum_{\mathrm{j}=0}^{\mathrm{q}}{\Delta \mathrm{RECE}}_{it-j}^{-}+\varnothing 5\sum_{\mathrm{j}=0}^{\mathrm{q}}\Delta {\mathrm{ELE}}_{\mathrm{it}-\mathrm{j}}+\varnothing 6\sum_{\mathrm{j}=0}^{\mathrm{q}}\Delta {\mathrm{IND}}_{\mathrm{it}-\mathrm{j}}+\varnothing 7\sum_{\mathrm{j}=0}^{\mathrm{q}}\Delta {\mathrm{HC}}_{\mathrm{it}-\mathrm{j}}+\uplambda {1\mathrm{LCF}}_{\mathrm{it}-1}+\uplambda {2\mathrm{GDP}}_{\mathrm{it}-1}+ {\uplambda 3\mathrm{RECE}}_{it-j}^{+}+{\uplambda 4\mathrm{RECE}}_{it-j}^{-}+\uplambda {5\mathrm{ELE}}_{\mathrm{it}-1}+\uplambda {6\mathrm{IND}}_{\mathrm{it}-1}+\uplambda {7\mathrm{HC}}_{\mathrm{it}-1} +\mathrm{ \varphi }{\mathrm{ECT}}_{\mathrm{t}-1}+{\varepsilon }_{it}$$

**Figure 1:**
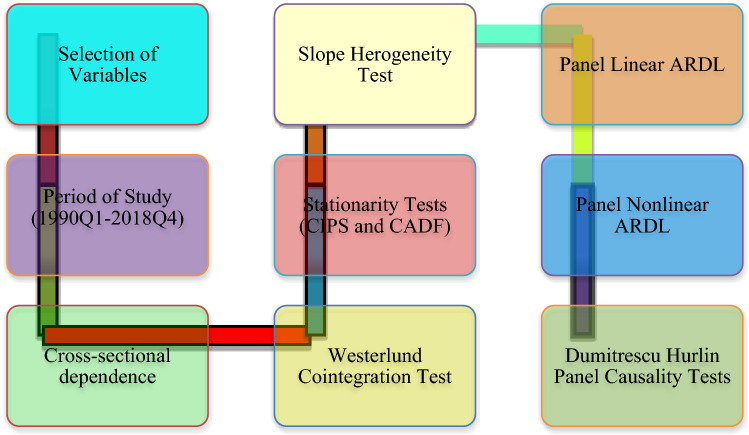
Flow of the study.

The PNARDL method does not show any causal linkage among the selected variables. Hence, the study utilized the Dumitrescu-Hurlin causality technique to assess the causal connection between the LCF and the regressors (LCF, EG, RECE, IND, HC, ELE). This assessment is widely accepted as a sophisticated method for causality, considering the size and the cross-section's time dimension. This approach also is essential in our study to make policy recommendations. The flow of analysis is presented in Fig. 1.

## Empırıcal results

### Outcomes of cross‑sectional dependence and unit roots assessments

First, the research utilized the Cross-sectional dependence (CD) technique to investigate whether or not CD exists. The outcomes of the CD test are presented in Table 1, which explains that the *p*-value is significant at (1%), implying the rejection of *the H0* hypothesis. Furthermore, the current work employed CIPS and CADF techniques to assess the stationary levels amid the concentrated variables. The CIPS and CADF outcomes are illustrated in Table 1, which shows that all the selected variables with the exemption of IND are not stationary at the level. However, at the first difference, they became stationary. However, these techniques proved that all explored parameters are I(1).

**Table 1:** CD and CIPS, and CADF Test outcomes.

Variables	CD-outcomes	*p*-value	CIPS-outcomes		CADF-outcomes	
			*I(0)*	*I(I)*	*I(0)*	*I(I)*
LCF	15.290*	0.0000	− 1.874	− 4.740*	− 2.580	− 4.740*
EG	18.927*	0.0000	− 1.620	− 3.430*	− 0.948	− 3.430*
RECE	9.8274*	0.0000	− 0.873	− 5.067*	− 1.803	− 5.067*
IND	15.067*	0.0000	− 3.207*	–	− 3.372*	–
HC	19.894*	0.0011	− 1.639	− 5.703*	− 2.302	− 5.368*
ELE	12.097*	0.0000	− 1.625	− 5.468*	− 2.494	− 5.510*

*Denotes a 1% level of significance.

The study utilized the slope heterogeneity (SH) test. The outcomes of SH test (Table 2) reported that the values of both "delta $\hat{\Delta }$" and adjusted "delta $\hat{\Delta }_{{{\text{Adj}}}}$" are significant at 1% level. These outcomes proved that there is slope heterogeneity in the employed model, which can be attributed to the BRICS-T region not having the same economic condition.

**Table 2:** Findings of SH test.

$\hat{\Delta }$	*P* value	$\hat{\Delta }_{{{\text{Adj}}}}$	*P* value
7.441*	0.000	8.362*	0.000

*Denotes a 1% level of significance.

### Co-integration test results

As previously noted, if the unit root assessments affirm that the examined data are stationary, we proceed to assess the co-integration among the tested variables. The current study utilized Westerlund co-integration to capture the long-run association between the variables. The outcomes of co-integration technique are depicted in Table 3, which indicates that the Ho hypothesis of "no co-integration" is rejected. Thus, this test findings provide evidence of co-integration among the examined parameters.

**Table 3:** Westerlund cointegration.

Statistics	Value	Z-value	*p*-value	Robust. *P* value
Gt	− 3.127	− 2.511	0.006	0.000
Ga	− 8.802	2.694	0.942	0.000
Pt	− 16.974	− 10.863	0.000	0.060
Pa	− 11.968	1.338	0.903	0.000

### Linear ARDL Results

The outcomes of the symmetric ARDL approach are displayed in Table 4. This approach showed that an upsurge in the level of RELE promotes LCF in BRICS-T region. The study illustrated that a one percent increase in RELE enhanced the LCF by 0.123% and 0.017% in long and short periods. On the other hand, the outcomes reported that an upsurge in the level of ELE has an adverse influence on the environmental neutrality in BRICS-T region. The results demonstrated that a 1 percent increase in ELE led to a significant decline in the LCF by 0.2091 and 0.148 in long and short periods.

**Table 4:** Panel ARDL.

	Long-run			Short-run		
	Coefficient,	t-Stat	Prob,	Coefficient,	t-Stat	Prob,
${\text{EG}}$	− 1.9106*	− 3.0367	0.0029	0.4886	1.1236	0.2633
${\text{ELE}}$	− 0.2091**	− 2.2827	0.0218	− 0.2091**	− 2.2827	0.0218
${\text{IND}}$	− 1.9512*	− 5.7733	0.0000	− 0.5472**	− 2.0552	0.0420
${\text{HC}}$	1.4005*	3.0868	0.0025	1.4005*	3.0868	0.0025
RECE	0.1230**	1.9976	0.0480	0.017	0.3258	0.7451
ECT (− 1)				− 0.3679*	− 3.0709	0.0027
C	− 4.9141	− 1.162584	0.2472			

$\Delta$denote short-run. Significance level of 10%, 5% and 1% are shown by ***, ** and * respectively.

Moreover, the findings reported that economic growth in BRICS-T nations adversely affected ecological quality in BRICS-T in both the long and short term. The outcomes reported that a 1% rise in economic growth led to a mitigation of the LCF by 1.901% and 0.4886% in long and short periods, respectively. These findings align with the study of^22^, who scrutinized the real growth-environment connection in the BRICS region using data between 1990 and 2018. Likewise, the study showed that an increase in industrialisation in BRICS-T nations negatively affected the ecological quality in BRICS-T. The findings from this work suggested that a 1 percent upsurge in industrialisation mitigates the LCF by 1.9512% in the long term and 0.5472% in the short term, respectively. The empirical outcomes also showed that improving the human capital in BRICS-T nations positively affected the ecological neutrality in BRICS-T in the long term. In contrast, in the short period, this impact is insignificant.

### Nonlinear ARDL results

**Figure 2:**
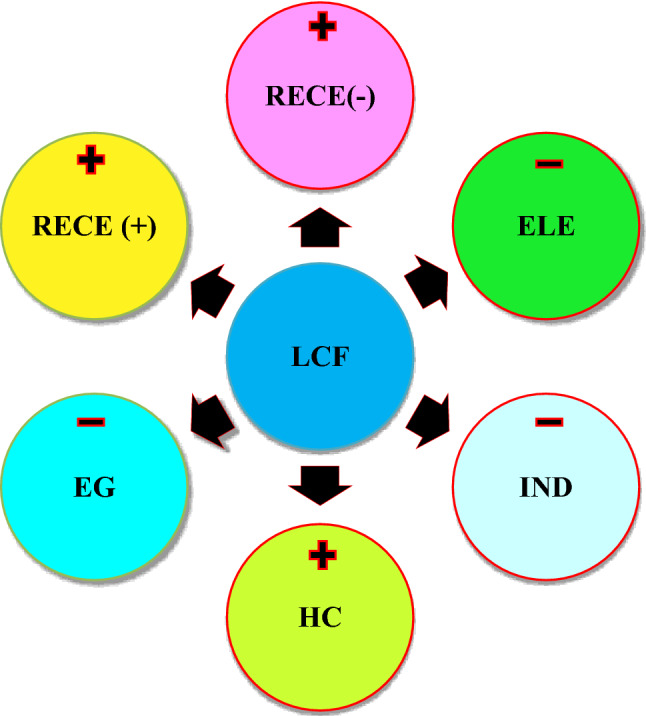
Summary of the linear and nonlinear ARDL.

The study explored the asymmetric linkage the dependent and independent variables using the nonlinear autoregressive distributed lag (NARDL). The NARDL outcomes reported that a 1% positive increase in RECE promotes the LCF by 0.0418% in the long term and 0.0176% in the short term. Furthermore, the findings showed a 1% negative shift in RECE increases LCF by 0.0770% (long-term) and 0.0504% (short-term). In contrast, the outcomes from the nonlinear model test demonstrated that a 1% upsurge in ELE influenced LCF positively in the long and short term. The outcomes revealed that a 1% positive shift in ELE mitigates LCF by 4.508% and 1.2103% in long and short periods, respectively. Meanwhile, an upsurge in IND contributes to a decrease in LCF. This result illustrated that a 1% shift in industrialisation decreased LCF by 0.5385% in the long run and 0.1935%% in the short period, respectively. Finally, HC contributes positively to LCF. This illustrates that a 1% upsurge in HC increase LCF by 1.5075% in the long term and 0.1714% in the short term. The effect of economic expansion is positive and significant in the long and short term. Specifically, a 1% in EG decreases LCF by 0.871% in the long-term and 0.669% in the short-term. Table 5 presented the Panel Nonlinear ARDL. Figure 2 presents the summary of findings from the linear and nonlinear ARDL.

**Table 5:** Panel nonlinear ARDL.

	Long-run			Short-run		
	Coefficient,	t-Stat	Prob,	Coefficient	t-Stat	Prob
${\text{EG}}$	− 0.8711**	− 2.4281	0.0176	− 0.6694**	− 2.0845	0.0267
${\text{ELE}}$	− 4.5080*	− 5.6060	0.0000	1.2103	1.5411	0.1276
${\text{IND}}$	− 0.5385***	− 1.7381	0.0864	− 0.1935**	− 2.3658	0.0196
${\text{HC}}$	1.5075***	1.8319	0.0710	0.1714	1.6089	0.1119
${\text{RECE}}^{ + }$	0.0418*	3.1797	0.0022	− 0.0695*	− 3.9494	0.0000
${\text{RECE}}^{ - }$	0.0070*	2.9542	0.0042	0.0504*	4.6346	0.0000
ECT (− 1)	–	–		− 0.8818*	− 6.5904	0.0000
C	1.9718	1.0176	0.3124	–		

Significance level of 10%, 5% and 1% are shown by ***, ** and * respectively.

### Dumitrescu Hurlin panel results

To study the causal interconnection between the selected variables, we utilized the Dumitrescu Hurlin panel causality test. The findings of this test are displayed in Table 6, which shows that there is a bi-directional causal association between EG and LCF. In addition, the results showed that there is un-directional causality from electricity consumption, from non-renewable energy, industrialisation, and human capital to LCF. At the same time, the outcomes demonstrated that there is un-directional causality from LCF to RECE. These findings affirm the findings of ARDL and NARDL testing approaches. In this context, the findings reinforce the significant influence of economic growth, use of electricity from renewable and non-renewable sources, and human capital on the LCF of BRICS-T states.

**Table 6:** Dumitrescu Hurlin panel causality tests.

Null hypothesis:	W-stat	Zbar-stat	Prob
EG $\ne$ LCF	6.53748*	8.12439	0.0000
LCF $\ne$ EG	8.21340*	10.6225	0.0000
ELEC $\ne$ LCF	6.29120*	7.75730	0.0000
LCF $\ne$ ELEC	0.32080	− 1.14201	0.2534
IND $\ne$ LCF	8.65406*	11.2793	0.0000
LCF $\ne$ IND	1.45776	0.55111	0.5816
HC $\ne$ LCF	4.30046*	4.76450	0.0000
LCF $\ne$ HC	2.08019	1.46992	0.1416
${\text{RECE}}^{ + }$ $\ne$ LCF	9.51820*	12.5674	0.0000
LCF $\ne {\text{RECE}}^{ + }$	0.86021	− 0.33858	0.7349
LCF $\ne {\text{RECE}}^{ - }$	2.42638**	1.99651	0.0459
$${\text{RECE}}^{ - }$$$$\ne$$ LCF	0.99036	− 0.14398	0.8855

Significance levels of 5% and 1% are shown by **and *, respectively.

### Discussion of findings

The study finding shows that the growth trajectory of the BRICS-T nations is not sustainable as shown by the negative connection between ecological quality and economic growth. This position reinforced the EKC hypothesis where developing nations such as BRICS-T economies focused more on economic expansion while paying less attention to ecological sustainability. The findings are essential for assisting in creating and using green economic growth initiatives in the BRICS nations, where economic growth is steadily accelerating. For example, energy efficiency strategies have been advocated in BRICS nations, where energy usage is expected to rise over the next two decades in order to maintain and advance economic growth without generating excessive levels of ecological devastation^2,35,36^. The studies of^37,38^, and^39^ reported similar findings by establishing the emissions-increasing effects of economic growth.

Furthermore, the process of industrialisation damages the environment by producing additional contaminants. This is because resources have been ruthlessly exploited to further industrialisation without considering ecological concerns. Moreover,^40^ contends that variables, including industrialisation, can positively impact CO_2_ emissions while income levels are rising. Likewise, given the increasing need for economic development and employment prospects, the negative externalities related to environmental deterioration in BRICS nations will continue to outweigh the positive externalities connected with industrialisation. For both emerging and developed nations worldwide, a scenario like this leads to increased energy usage, which has a knock-on effect of increasing emission levels at both the sectoral and individual level. Also, growing family incomes influence consumer demand to the point that expansion in the manufacturing industry fuels additional industrial expansion, which raises ecological deterioration. Moreover,^32^ recommended adopting more green energy options to lower expected emission levels in order to avoid such a situation. The pressing need for stricter industrial ecological rules to prevent increasing CO_2_ emissions was also backed up by prior studies^41^^,^^42^^,^^43^.

Moreover, a positive increase in electricity consumption from non-renewable energy increases ecological deterioration by decreasing load capacity factor. The reason is that the non-renewable energy sources are fossil fuel based and produce polluting elements while consumed. This finding is in line with^18^ and the results of^40^. On the flip side, the electricity consumption from renewable energy decreases ecological deterioration by increasing load capacity factor. Also, renewable energy is secure and long-lasting enough to provide environmental benefits without slowing down the pace of progress. Renewable energy sources are also sufficient and environmentally benign, and they cause less ecological damage.

Moreover, a positive increase in electricity consumption from non-renewable energy increases ecological deterioration by decreasing the load capacity factor. The explanation is that non-renewable sources of energy are based on fossil fuels and release pollutants when used. This discovery is consistent with the prior studies^44,45^. On the other hand, by raising the load capacity factor, using electricity generated from renewable sources slows down ecological deterioration. Also, renewable energy is secure and long-lasting enough to provide environmental benefits without slowing down the pace of progress. This outcome is also validated by prior studies^46,47^. In addition to being sufficient and environmentally benign, renewable energy sources emit less CO_2_ emissions. Moreover, the renewable energy use of BRICS-T members only accounted for 16 percent of total energy use. Besides, the BRICS-T members consume around 40% of the total energy in the world and release approximately 40% of global carbon emissions. In this context, the highest electricity consumption from renewable energy in BRICS-T countries is seen in Brazil, which currently hosts one of the cleanest energy matrices of the industrialized world, with approximately 89% of all electricity supply in this country coming from renewable energy sources. In contrast, China and South Africa have the lowest rates of renewable energy use. In comparison, the highest carbon emitters in BRICS-T are seen in China and South Africa, which emits more greenhouse gas than the entire developed world combined, whereas Bazil has the lowest.

The human capital is positively associated with ecological quality in the BRICS countries. These findings are comparable to the studies of^11,37,48–50^. Thanks to their enhanced human capital, the BRICS nations can effectively apply green technological approaches to change the economic architecture to renewable energy sources. Hence, one important aspect influencing ecological quality is human capital.

## Conclusions and policy recommendations

### Conclusion

This paper evaluates the impact of electricity consumption from non-renewable and renewable on ecological sustainability (proxied by load capacity factor) for BRICS-T nations from 1990 to 2018. The study used linear and nonlinear ARDL techniques to evaluate these associations. The paper used linear and nonlinear autoregressive distributed lag approaches to explore these associations. The results of the Westerlund co-integration show long-run co-integration between LCF and the independent variables. The outcomes from the nonlinear approach(NARDL) reported that a positive increase in electricity consumption from renewables promotes the LCF. In contrast, a negative move in electricity consumption from renewables mitigates the LCF. In comparison, the outcomes from the nonlinear approach demonstrated that an increase in electricity consumption from non-renewable has an adverse influence on LCF. These findings suggest that non-renewable electricity energy mitigates ecological sustainability in the BRICS-T nations while renewable electricity energy promotes ecological sustainability.

### Policy recommendations

The study advocates the following policy actions based on our empirical results.

- Emerging economies such as the BRICS nations must invest in the crucial technological and infrastructure initiatives that promote sustainable industrialisation, boost economic development, lower carbon dioxide emissions, and improve regulatory frameworks. Proper management of the chemical and heavy industrial sectors is crucial, especially since they not only exacerbate environmental problems but also play a significant role in boosting the manufacturing capacity of emerging nations. Effective energy and ecological regulations can also boost increased levels of foreign investment, improved energy security via diversification, and increased technological innovation, all of which guarantee that more sustainable forms of economic progress are accomplished at a coherent level across the nations. In response, policymakers should concentrate on regulatory change in high-emissions sectors by funding projects or offering tax breaks to businesses that use greener technologies. Also, the governments of the BRICS countries should promote measures that enable a new low-carbon reform agenda to emerge in addition to increasing research and development investment in cleaner technologies.
- The BRICS nations ought to enact stronger energy-use regulations that cut emissions and gradually phase out investments in and dependence on fossil fuel energies. By burning fossil fuels, non-renewable energy reduces ecological quality. The usage of non-renewable energy should be minimized as much as feasible in order to decrease contamination. Using non-renewable energy less would safeguard the ecosystem and guarantee sustainable supplies. To formulate successful policy initiatives and improve ecological quality, sincere and daring efforts are required.
- Policies that aim to improve ecological quality must also focus on improving energy efficiency and restructuring the industrial base. Technological improvements, expenditures on energy-efficient machinery, and adjustments to the energy structure might achieve this. Specific incentives should be provided to help the BRICS nations improve their energy efficiency systems. As a result, it is important to raise taxes on the use of fossil fuels in order to lessen the hazard of ecological deterioration and to allay fears about climate change. Besides that, the BRICS nations should, wherever feasible, ratify and uphold the essential tenets of international agreements like the Paris Agreement.
- Improving contemporary technologies and more effective energy utilization will improve the BRICS nations' environmental conditions. BRICS nations' governments and other important stakeholders, including companies and local communities, are urged to prioritize non-renewable energy research while also putting in place the necessary frameworks for a policy that may increase both their efficiency and affordability. More work must be accomplished to enable the efficient transfer of skills, information, and valuable technologies that BRICS nations need to flourish in a globe that is becoming more ecologically conscious in light of establishing a worldwide 2050 zero-carbon target.
- Also, the mentioned BRICS nations must put eco-friendly policies into practice via strengthened human capital to deal with environmental deterioration. When combined with eco-friendly technologies, such tactics will provide the BRICS nations the push they need to change their economies to rely more on renewable energy sources. Enhancing educational achievement would boost domestic output and societal well-being while also enhancing ecological integrity. So, while formulating policy for environmental quality, human capital must be taken into account.

### Caveat of study and future path

The study has some drawbacks. Firstly, we assessed the linkage among the focused variables period from 1990–2018. However, the present paper’s limitation is the unavailability of some data sets after 2018 for load capacity factor. Second, the present paper employed a nonlinear technique to evaluate the connection between the employed variables in the BRICS-T region. Future empirical works may focus on assessing the effect of human capital on the load capacity factor by using different approaches and other economies.

## Data Availability

The datasets used and/or analysed during the current study available from the corresponding author on reasonable request.

## Abbreviations

BRICS-T: Brazil, Russia, India, China, South Africa and Turkey
ARDL: Autoregressive distributed lag
GDP: Gross domestic product
IND: Industrialization
LCF: Load capacity factor
ELE: Electricity consumption (ELE) from non-renewable
RECE: Electricity consumption (RECE) from renewable
HC: Human capital
SH: Slope heterogeneity
PNARDL: Panel nonlinear ARDL
ARDL: Autoregressive distributed Lag
ECT: Error correction-term
*ε*_it_: Error term
FMOLS: Fully modified-OLS
CO_2_: Carbon emissions
AMG: Augmented mean group
CSD: Cross-sectional dependence
PMG: Pooled mean group

## References

[1] Shahbaz M, Sbia R, Hamdi H, Ozturk I (2014). Economic growth, electricity consumption, urbanization and environmental degradation relationship in United Arab Emirates. Ecol. Ind..

[2] Alola AA, Bekun FV, Sarkodie SA (2019). Dynamic impact of trade policy, economic growth, fertility rate, renewable and non-renewable energy consumption on ecological footprint in Europe. Sci. Total Environ..

[3] Ozturk I, Acaravci A (2016). Energy consumption, CO2 emissions, economic growth, and foreign trade relationship in Cyprus and Malta. Energy Sour. Part B.

[4] Balcilar M, Ozdemir ZA, Tunçsiper B, Ozdemir H, Shahbaz M (2020). On the nexus among carbon dioxide emissions, energy consumption and economic growth in G-7 countries: New insights from the historical decomposition approach. Environ. Dev. Sustain..

[5] Abbasi KR, Shahbaz M, Jiao Z, Tufail M (2021). How energy consumption, industrial growth, urbanization, and CO_2_ emissions affect economic growth in Pakistan? A novel dynamic ARDL simulations approach. Energy.

[6] Appiah M, Gyamfi BA, Adebayo TS, Bekun FV (2022). Do financial development, foreign direct investment, and economic growth enhance industrial development? Fresh evidence from Sub-Sahara African countries. Port. Econ. J..

[7] Costantini V, Crespi F, Marin G, Paglialunga E (2017). Eco-innovation, sustainable supply chains and environmental performance in European industries11We gratefully acknowledge the support by the European Union’s Horizon 2020 research and innovation programme under grant agreement No. 649186—ISIGrowth. The comments and suggestions by three anonymous referees are also acknowledged. The usual disclaimers apply. J. Clean. Prod..

[8] Adebayo TS (2022). Environmental consequences of fossil fuel in Spain amidst renewable energy consumption: A new insights from the wavelet-based granger causality approach. Int. J. Sustain. Dev. World Ecol..

[9] Ağa M, Adebayo TS, Ullah S, Kartal MT, Ali K, Pata UK (2023). Endorsing sustainable development in BRICS: The role of technological innovation, renewable energy consumption, and natural resources in limiting carbon emission. Sci. Total Environ..

[10] Gyamfi BA, Agozie DQ, Bekun FV (2022). Can technological innovation, foreign direct investment and natural resources ease some burden for the BRICS economies within current industrial era?. Technol. Soc..

[11] Hao L-N, Umar M, Khan Z, Ali W (2021). Green growth and low carbon emission in G7 countries: How critical the network of environmental taxes, renewable energy and human capital is?. Sci. Total Environ..

[12] Khan Z, Malik MY, Latif K, Jiao Z (2020). Heterogeneous effect of eco-innovation and human capital on renewable & non-renewable energy consumption: Disaggregate analysis for G-7 countries. Energy.

[13] Y. Shin, B. Yu, and M. Greenwood-Nimmo, (2014) modelling asymmetric co-integration and dynamic multipliers in a nonlinear ARDL framework. In: *Festschrift in Honor of Peter Schmidt: Econometric Methods and Applications*, R. C. Sickles and W. C. Horrace, Eds. New York, NY: Springer, pp. 281–314. 10.1007/978-1-4899-8008-3_9.

[14] Grossman GM, Krueger AB (1995). Economic growth and the environment*. Q. J. Econ..

[15] Salahuddin M, Alam K, Ozturk I (2016). The effects of Internet usage and economic growth on CO2 emissions in OECD countries: A panel investigation. Renew. Sustain. Energy Rev..

[16] Shahbaz M, Hye QMA, Tiwari AK, Leitão NC (2013). Economic growth, energy consumption, financial development, international trade and CO_2_ emissions in Indonesia. Renew. Sustain. Energy Rev..

[17] Borhan H, Ahmed EM, Hitam M (2012). The impact of Co_2_ on economic growth in Asean 8. Proc. Soc. Behave. Sci..

[18] Awosusi AA, Altuntaş M, Agyekum EB, Zawbaa HM, Kamel S (2022). The dynamic impact of biomass and natural resources on ecological footprint in BRICS economies: A quantile regression evidence. Energy Rep..

[19] Xu D (2022). Load capacity factor and financial globalization in brazil: The role of renewable energy and urbanization. Front. Environ. Sci..

[20] Usman O, Iortile IB, Ike GN (2020). Enhancing sustainable electricity consumption in a large ecological reserve–based country: the role of democracy, ecological footprint, economic growth, and globalisation in Brazil. Environ. Sci. Pollut. Res..

[21] Adebayo TS, Oladipupo SD, Adeshola I, Rjoub H (2022). Wavelet analysis of impact of renewable energy consumption and technological innovation on CO_2_ emissions: Evidence from Portugal. Environ. Sci. Pollut. Res..

[22] Adebayo TS, Samour A (2023). Renewable energy, fiscal policy and load capacity factor in BRICS countries: Novel findings from panel nonlinear ARDL model. Environ. Dev. Sustain..

[23] Akadiri SS, Asuzu OC, Onuogu IC, Oji-Okoro I (2022). Testing the role of economic complexity on the ecological footprint in China: A nonparametric causality-in-quantiles approach. Energy Environ..

[24] Adebayo TS, Akadiri SS, Asuzu OC, Pennap NH, Sadiq-Bamgbopa Y (2022). Impact of tourist arrivals on environmental quality: A way towards environmental sustainability targets. Curr. Issues. Tourism.

[25] Alola AA (2019). The trilemma of trade, monetary and immigration policies in the United States: Accounting for environmental sustainability. Sci. Total. Environ..

[26] Pata UK, Samour A (2023). Assessing the role of the insurance market and renewable energy in the load capacity factor of OECD countries. Environ. Sci. Pollut. Res..

[27] Li S, Samour A, Irfan M, Ali M (2023). Role of renewable energy and fiscal policy on trade adjusted carbon emissions: Evaluating the role of environmental policy stringency. Renew. Energy.

[28] Rahman MM, Nepal R, Alam K (2021). Impacts of human capital, exports, economic growth and energy consumption on CO_2_ emissions of a cross-sectionally dependent panel: Evidence from the newly industrialized countries (NICs). Environ. Sci. Policy.

[29] Mahmood N, Wang Z, Hassan ST (2019). Renewable energy, economic growth, human capital, and CO_2_ emission: An empirical analysis. Environ. Sci. Pollut. Res..

[30] Appiah M, Li M, Onifade ST, Gyamfi BA (2022). Investigating institutional quality and carbon mitigation drive in Sub-Saharan Africa: Are growth levels, energy use, population, and industrialization consequential factors?. Energy Environ.

[31] Rahman MdH, Majumder SC (2022). Empirical analysis of the feasible solution to mitigate the CO_2_ emission: Evidence from next-11 countries. Environ. Sci. Pollut. Res..

[32] Ahmed N, Ahmad M, Ahmed M (2022). Combined role of industrialization and urbanization in determining carbon neutrality: Empirical story of Pakistan. Environ .Sci. Pollut. Res..

[33] Li Y, Rui W, Qingmin Z, Zhaojie X (2022). Technological advancement and industrialization path of Sinopec in carbon capture, utilization and storage China. Energy Geosci..

[34] M. H. Pesaran, Y. Shin, and others, (1995) An autoregressive distributed lag modelling approach to co-integration analysis.

[35] Acheampong AO (2018). Economic growth, CO_2_ emissions and energy consumption: What causes what and where?. Energy Econ..

[36] Adedoyin FF, Gumede MI, Bekun FV, Etokakpan MU, Balsalobre-lorente D (2020). Modelling coal rent, economic growth and CO2 emissions: Does regulatory quality matter in BRICS economies?”. Sci. Total Environ..

[37] Ahmed Z, Asghar MM, Malik MN, Nawaz K (2020). Moving towards a sustainable environment: The dynamic linkage between natural resources, human capital, urbanization, economic growth, and ecological footprint in China. Resour. Policy.

[38] Baniya B, Aryal PP (2022). Nepal’s domestic material consumption—projection and causal impact of external financial inflows, services value-added, population, and economic growth. Environ. Sci. Pollut. Res..

[39] Bekun FV, Emir F, Sarkodie SA (2019). Another look at the relationship between energy consumption, carbon dioxide emissions, and economic growth in South Africa. Sci. Total Environ..

[40] Alola AA, Adebayo TS (2022). Are green resource productivity and environmental technologies the face of environmental sustainability in the Nordic region?. Sustain. Dev..

[41] Chikezie Ekwueme D, Lasisi TT, Eluwole KK (2022). Environmental sustainability in Asian countries: Understanding the criticality of economic growth, industrialization, tourism import, and energy use. Energy Environ..

[42] Wang W, Rehman MA, Fahad S (2022). The dynamic influence of renewable energy, trade openness, and industrialization on the sustainable environment in G-7 economies. Renew. Energy.

[43] Ullah S, Ozturk I, Usman A, Majeed MT, Akhtar P (2020). On the asymmetric effects of premature deindustrialization on CO_2_ emissions: Evidence from Pakistan. Environ. Sci. Pollut. Res..

[44] Balsalobre-Lorente D, Shahbaz M, Roubaud D, Farhani S (2018). How economic growth, renewable electricity and natural resources contribute to CO_2_ emissions?. Energy Policy.

[45] Bilgili F, Ozturk I, Kocak E, Kuskaya S, Cingoz A (2022). The nexus between access to electricity and CO_2_ damage in Asian Countries: The evidence from quantile regression models. Energy Build..

[46] Çıtak F, Şişman MY, Bağcı B (2021). Nexus between disaggregated electricity consumption and CO2 emissions in Turkey: New evidence from quantile-on-quantile approach. Environ. Ecol. Stat..

[47] Farhani S, Shahbaz M (2014). What role of renewable and non-renewable electricity consumption and output is needed to initially mitigate CO_2_ emissions in MENA region?. Renew. Sustain. Energy Rev..

[48] Ahmad M, Ahmed Z, Yang X, Hussain N, Sinha A (2022). Financial development and environmental degradation: Do human capital and institutional quality make a difference?. Gondwana. Res..

[49] Jahanger A (2022). Impact of globalization on CO_2_ emissions based on EKC hypothesis in developing world: The moderating role of human capital. Environ. Sci. Pollut. Res..

[50] Pata UK, Samour A (2022). Do renewable and nuclear energy enhance environmental quality in France? A new EKC approach with the load capacity factor. Progress in Nuclear Energy.

